# Effect of Manure and Compost on the Phytostabilization Potential of Heavy Metals by the Halophytic Plant Wavy-Leaved Saltbush

**DOI:** 10.3390/plants10102176

**Published:** 2021-10-14

**Authors:** Jianjian Li, Yajun Chang, Arwa Abdulkreem AL-Huqail, Zheli Ding, Mohammad S. Al-Harbi, Esmat F. Ali, Amany H. A. Abeed, Saudi A. Rekaby, Mamdouh A. Eissa, Adel M. Ghoneim, Suzan A. Tammam

**Affiliations:** 1 Institute of Botany, Jiangsu Province and Chinese Academy of Sciences, Nanjing Botanical Garden, Mem. Sun Yat-Sen, Nanjing 210014, China; lijianjian2015@cnbg.net (J.L.); changyj@cnbg.net (Y.C.); 2 Department of Biology, College of Science, Princess Nourah Bint Abdulrahman University, Riyadh 13324-8824, Saudi Arabia; 3 Haikou Experimental Station, Chinese Academy of Tropical Agricultural Sciences (CATAS), Haikou 571101, China; dingzheli@zju.edu.cn; 4 Department of Biology, College of Science, Taif University, P.O. Box 11099, Taif 21944, Saudi Arabia; mharbi@tu.edu.sa (M.S.A.-H.); a.esmat@tu.edu.sa (E.F.A.); 5 Department of Botany and Microbiology, Faculty of Science, Assiut University, Assiut 71516, Egypt; dramany2015@aun.edu.eg (A.H.A.A.); susantammam@yahoo.com (S.A.T.); 6 Department of Soils and Water, Faculty of Agriculture, Al-Azhar University (Assiut Branch), Assiut 71524, Egypt; saudirekaby.4419@azhar.edu.eg; 7 Department of Soils and Water, Faculty of Agriculture, Assiut University, Assiut 71526, Egypt; 8 Agricultural Research Center, Field Crops Research Institute, Giza 12112, Egypt; adelrrtc.ghoneim@gmail.com; 9 Biology Department, Faculty of Science, Al-Baha University, Al-Baha 65779-77388, Saudi Arabia

**Keywords:** contaminated soils, *Atriplex*, halophytic plants, organic amendments, phytoremediation

## Abstract

This study aimed to use organic fertilizers, e.g., compost and manures, and a halophytic plant [wavy-leaved saltbush (*Atriplex undulata*)] to remediate an agricultural soil polluted with toxic elements. Compost or manure (1% *w*/*w*) was added to a polluted soil in a pot trial. The application of the organic fertilizer, whether compost or manure, led to a significant improvement in the growth of the tested plant. From the physiological point of view, the application of organic fertilizers to polluted soil significantly increased the content of chlorophyll, carotenoid, and proline and, furthermore, led to a clear decrease in malondialdehyde (MDA) in the plant leaves. The highest significant values of organic carbon in the polluted soil (SOC) and cation exchange capacity (CEC) were found for the soil amended by compost and planted with wavy-leaved saltbush. Manure significantly reduced the soil pH to 7.52. Compost significantly decreased Zn, Cu, Cd, and Pb availability by 19, 8, 12, and 13%, respectively, compared to the control. On the other hand, manure increased Zn, Cu, Cd, and Pb availability by 8, 15, 18, and 14%, respectively. Compost and manure reduced the bioconcentration factor (BCF) and translocation factor (TF) of Cd and Pb. Compost was more effective in increasing the phytostabilization of toxic metals by wavy-leaved saltbush plants compared to manure. The results of the current study confirm that the application of non-decomposed organic fertilizers to polluted soils increases the risk of pollution of the ecosystem with toxic elements. The cultivation of contaminated soils with halophytic plants with the addition of aged organic materials, e. g., compost, is an effective strategy to reduce the spreading of toxic metals in the ecosystem, thus mitigating their introduction into the food chain.

## 1. Introduction

In recent decades, soil contamination with heavy metals has raised concern due to its negative impact on environmental systems and the possibility of heavy metals bioaccumulation in the food chain [1]. The soil is a main sink for toxic elements from different human activities and a main source of food contamination [2,3]. In addition, pollution of agricultural land with heavy metals leads to the deterioration of its properties and suitability for plant production [3,4,5]. The deterioration of soil properties will eventually lead to a decrease in the productivity of crops and in their suitability for human consumption [3,4]. Toxic elements such as arsenic (As), copper (Cu), cadmium (Cd), chromium (Cr), lead (Pb), and zinc (Zn) are the most diffuse environmental polluting elements [5]. Agriculture lands are a nonrenewable resource on earth, so the conservation of contaminated soils is vital for sustainable development [3]. About 20 million hectares of world agricultural lands have heavy metal levels higher than the permissible limits [4,6].

There are many methods to remediate a polluted soil, but ecologists prefer to use biological methods to reduce pollution and preserve the ecosystem [3,6]. The use of plants as an environmentally friendly method to remediate polluted agriculture lands is a modern technology that is receiving wide attention from environmental scientists and governments [6]. The phytoremediation technology depends on the use of plants to eliminates contaminants from soils and water by adsorbing or degrading them [6,7]. Phytostabilization is one of the main phytoremediation strategies which involve the planting of suitable plant species able to immobilize heavy metals and prevent their accumulation in soils and ground water [3,8,9]. 

Some halophytic shrubs, especially those belong to the Chenopodiaceae family, are grown in saline arid conditions and are used to provide forage for livestock and the rehabilitation of saline soils [8,10]. *Atriplex* is one of the most important types of halophytic plants and is known as saltbush [11]. River saltbush (*Atriplex amnicola* Paul G. Wilson) is part of the Chenopodiaceae family and has been used in the remediation of polluted soils [8]. Halophytic plants, e.g., river saltbush, quail bush, and wavy-leaved saltbush, can be used in the remediation of metal-polluted soils [9]. Wavy-leaved saltbush (*Atriplex undulata*) is a plant of the *Atriplex* genus, but little is known about its phytoremediation efficiency. 

The first step to reduce metals pollution is the reduction of metal bioavailability in the contaminated soils [3]. Several organic fertilizers have been used for the immobilization of toxic elements in polluted lands [12,13,14]. Organic amendments are a source of plant nutrients and could remain the principal nutrients sources for the maintenance of soil fertility and quality [15]. Organic materials derived from plant and animal residues have many positive characteristics that can enhance soil quality and improve crop performance [16]. These materials can be particularly useful as amendments to severely degraded soils [11]. Compost is rich in phosphorus (P) and iron (Fe), which can immobilize toxic metals in polluted soil [17]. The impact of organic amendments on the bioavailability of toxic elements varies according to the degree of decomposition of the organic matter [18,19]. The oxidizable fraction of heavy metals which is bound to soil organic matter (SOM) is significantly influenced by the electrostatic complexation between the metal and SOM, which affects the toxicity of metals in contaminated soils [20]. The hydroxyl and phenolic groups in humic compounds are the chief ligands for toxic elements [21]. Humic substances form insoluble complexes with toxic metals and can decrease their bioavailability and pollution risks; therefore, organo–metal complexes can be an effective tool to decrease the environmental risk of heavy metals [22]. 

Compost differs in its chemical composition from manure that contains non-decomposing organic materials; therefore, it is expected that there will be a difference in their effects on the bioavailability of heavy metals. The current study was conducted to examine: (1) the efficiency of wavy-leaved saltbush plants in reducing the availability of Zn, Cu, Cd, and Pb, (2) the role of organic fertilizers, e.g., compost and manure, in increasing the ability of the halophytic plants to tolerate the toxic metal stress, and (3) the effects of compost and manure on the bioavailability, accumulation, and root-shoot transfer of Zn, Cu, Cd, and Pb.

## 2. Material and Methods

### 2.1. Soil Characterization

The soil of the current study was collected from a polluted private farm in Illwan, Assiut, Egypt. Untreated sewage wastewater was used in the irrigation of the experimental site for more than 60 years. The chemical and physical properties are shown in Table 1. The soil was air-dried and sieved through a 2 mm sieve. Soil texture was determined by the pipette method [23]. The soil pH was determined in a 1:2 (soil to water) suspension by a digital pH meter. Soil organic carbon was determined by the dichromate oxidation method as described by Wakley and Black [23]. CaCO_3_ was measured by the calcimeter method [23]. Soil salinity (EC) was estimated in a 1:2 soil-to-water extract by determining the electric conductivity [23]. The micro-Kjeldahl method was used to measure total nitrogen in soil [23], whereas 0.005 M DTPA (diethylenetriaminepentaacetic acid) was used to extract the available Zn, Cu, Cd, and Pb from soil samples [24]. The soil samples (2 g) were digested by HF-HNO3-HClO4 (1:1:1, *v*/*v*) in Teflon beakers to extract the total soil Zn, Cu, Cd, and Pb [23]. SRM 1547, a reference material, was determined for quality assurance and the results indicated that levels of Zn, Cu, Cd, and Pb are within the range of the reference material. All the chemical analysis was done in duplicate. 

### 2.2. Pot Experiments

Two series of pot experiments in a greenhouse (25 °C and 12 h light) were conducted to study the immobilization of toxic elements by *A. undulata*. The first series of experiment was conducted with the addition of compost and manure to the contaminated soil without any tested plants. The second series of experiment was performed in the same conditions with the inclusion of *A. undulata* plants. Plastic pots (25 cm in diameter and 20 cm in depth) were filled with 5 kg of soil. Two seedlings of *A. undulata* were planted in each pot. The pots were carefully watered near field capacity. Two types of organic fertilizers were used in the current study, i.e., compost and farmyard manure. Compost was obtained from plant material. The tested organic materials were added to the soil in the amount of 1% of soil weight before cultivation. The trials included a control treatment without fertilization. The same treatments were repeated in other pots without plants cultivation. The trials included 24 pots, 12 pots with plants, and 12 pots without plants. 

### 2.3. Characterization of Compost and Manure

A sample of compost and manure was burned in a muffle furnace at 600 °C for 6 h to determine the total organic content [23]. A mixture of H_2_SO_4_ and HClO_4_ (1:1 *v*/*v*) was used to digest a sample of compost and manure to determine Zn, Cu, Cd, Pb, and total nitrogen [23]. The pH of the organic materials was measured by a digital pH meter in a 1:10 suspension. The electrical conductivity (EC) was estimated in 1:10 extracts by electric conductivity measurement [23]. Table 2 shows the chemical analysis of compost and manure.

### 2.4. Plant Analysis

After 15 weeks of cultivation, the plants were harvested and separated into roots and shoots. The samples of roots and shoots were washed twice with tap water. A solution of 0.1 HCl and Tween 80 was used to remove the inorganic wastes from the plant samples, and then the samples were washed with distilled water. The plant samples were oven-dried at 70 °C for 48 h. The dried samples were ground and submitted to the acid digestion using a 2:1 HNO_3_/HClO_4_ mixture [23]. The concentration of toxic elements, e.g., Zn, Cu, Cd, and Pb, was measured by an atomic absorption spectrophotometer (AAS). Malondialdehyde (MDA) was measured by the method described by Madhava and Sresty [25]. Total chlorophylls and carotenoids were extracted from fresh leaves by acetone (80%) and then analyzed by a spectrophotometer [26].

### 2.5. Bioconcentration and Translocation Factors

The bioconcentration factor (BCF) is calculated by dividing metal in shoot by total metal in soil, according to Equation (1) [27]. BCF is determined to evaluate a plant ability to accumulate heavy metals; values of BCF higher than 1 indicate a higher accumulation capacity.

(1) $$\mathrm{BCF}=\mathrm{Metal}\;\mathrm{in}\;\mathrm{shoot}\;(\mathrm{mg}\;{\mathrm{kg}}^{-1})/\mathrm{Total}\;\mathrm{metal}\;\mathrm{in}\;\mathrm{soil}\;(\mathrm{mg}\;{\mathrm{kg}}^{-1})$$

The translocation factor (TF) is a parameter to evaluate the root–shoot transfer of metals and is calculated with Equation (2) [27].

(2) $$\mathrm{TF}=\mathrm{Shoot}\;\mathrm{metal}\;(\mathrm{mg}\;{\mathrm{kg}}^{-1})/\mathrm{Root}\;\mathrm{metal}\;(\mathrm{mg}\;{\mathrm{kg}}^{-1})$$

### 2.6. Statistical Analysis

The cultivated and non-cultivated pots were arranged in a Complete Blocks Design (CBD) with four replicates. One-way ANOVA was used to test the significance of the differences between the tested treatments. Duncan test was used to compare means. SPSS statistical software was used in all statistical analysis. 

## 3. Results 

### 3.1. Effect of Cultivation and Organic Fertilization on the Characteristics of Polluted Soil

Compost and manure were added to the polluted soil to explore their effects on the soil properties and the availability of Cd and Pb. The results for the studied characteristics of the cultivated and non-cultivated soils are reported in Table 3. The soil pH was affected significantly (*p* < 0.05) by cultivation and organic fertilization treatments. The pH values ranged between 7.52 and 7.90; the lowest significant value was found for the cultivated soil amended with manure, while the highest one was found for the control soil without cultivation. The addition of manure to the cultivated soil reduced the soil pH to 7.52 (Table 1 and Table 3). The highest significant values for soil organic carbon (SOC) and cation exchange capacity (CEC) were found for the soil amended by compost and cultivated with wavy-leaved saltbush plants.

The planting of metal-polluted land by wavy-leaved saltbush plants and the application of compost or manure significantly (*p* < 0.05) affected the studied properties and the bioavailability of Zn, Cu, Cd, and Pb. The cultivation of soil significantly minimized the bioavailability of Zn, Cu, Cd, and Pb. Compost decreased the availability of Zn, Cu, Cd, and Pb by 19, 8, 12, and 13% in the non-cultivated soil compared to the control, while manure increased these values by 8, 15, 18, and 14%. Compost addition to the planted soil caused a decrease in the bioavailability of Zn, Cu, Cd, and Pb by 10, 12, 24, and 15% compared to the control, while manure increased the availability of Zn, Cu, Cd, and Pb by 8, 10, 8. and 6%. The addition of compost decreased the bioavailability of Zn, Cu, Cd, and Pb; on the other hand, manure increased the availability of Zn, Cu, Cd, and Pb in the contaminated soil. The cultivation of soil with wavy-leaved saltbush plants decreased the availability of Zn, Cu, Cd, and Pb by 16, 27, 25, and 33% compared to the non-cultivated soil (over the manure and compost treatments). The availability of Zn, Cu, Cd, and Pb in the compost-amended and planted soil was decreased by 27, 29, 42, and 43% compared to the control without plants. Similarly, the availability of Zn, Cu, Cd, and Pb in the manure-amended and planted soil was decreased by 12, 27, 20, and 29% compared to the control without plants. Planting of the polluted soil by wavy-leaved saltbush plants was effective in reducing the availability of Zn, Cu, Cd, and Pb. Compost was more effective than manure in enhancing the immobilization of Zn, Cu, Cd, and Pb and in reducing metal bioavailability.

### 3.2. Effect of Compost and Manure on the Growth of Wavy-Leaved Saltbush Plants

The results related to the growth of wavy-leaved saltbush plants, plant length, root and shoot weight, number of leaves, and leaf area per plant, are reported in Table 4. The growth of wavy-leaved saltbush plants was affected significantly (*p* < 0.05) by the organic fertilization treatments. Compost or manure enhanced plant growth compared to the control. Compost addition increased plant length and root and shoot weight by 38, 50, and 38% in comparison with the control. Manure addition increased plant length and root and shoot weight by 27, 33, and 25% compared to the control soil. Compost increased the leaves number and area per plant by 60 and 50%, respectively, compared to the control. The application of manure increased the leaves number and area per plant by 50 and 38%, respectively, compared to the control soil.

### 3.3. Effect of Compost and Manure on Photosynthesis Pigments and Malondialdehyde (MDA) and Proline Content

**Figure 1:**
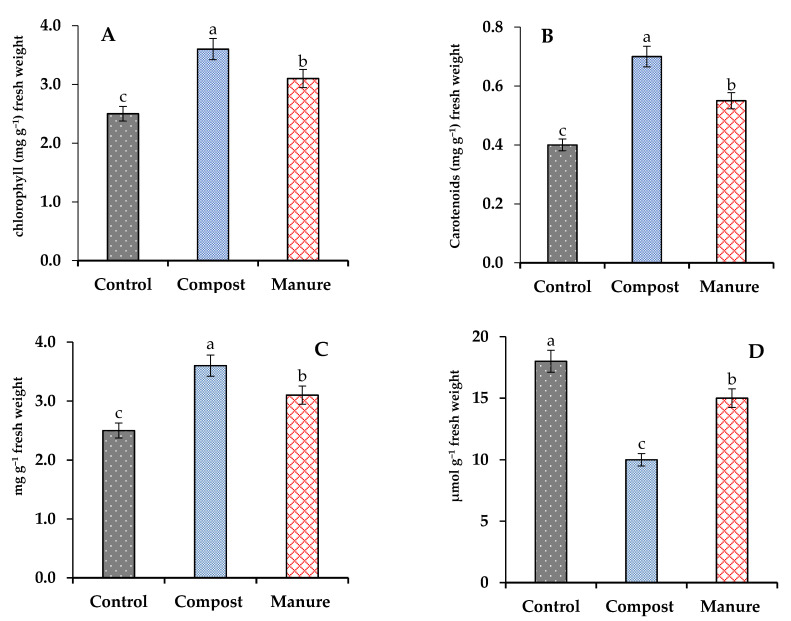
Effect of compost and manure on photosynthesis pigments (**A**) Total Chlorophyll, (**B**) Carotenoids), (**C**) Proline content, and (**D**) Malondialdehyde (MDA). Means (±standard deviation, *n* = 4) denoted by different letters are significantly different at *p* < 0.05. All the data are based on fresh weight.

The results related to the total chlorophyll, carotenoids, malondialdehyde (MDA), and proline content in wavy-leaved saltbush plants are shown in Figure 1. The content of some pigment in wavy-leaved saltbush plants was affected significantly (*p* < 0.05) by the organic fertilization treatments. Manure and compost increased total chlorophyll and carotenoids. Compost and manure increased chlorophylls by 44 and 24%, while these increments were 75 and 38% in the case of carotenoids.

The wavy-leaved saltbush plants cultivated on the control soil contained the highest significant values of MDA and the lowest significant value of proline. The application of compost and manure significantly (*p* < 0.05) decreased MDA by 44 and 17%, respectively, compared to the control, while the application of compost and manure increased proline by 44 and 24%, respectively, compared to the control. 

### 3.4. Cd and Pb Uptake and Translocation as Affected by Compost and Manure

**Figure 2:**
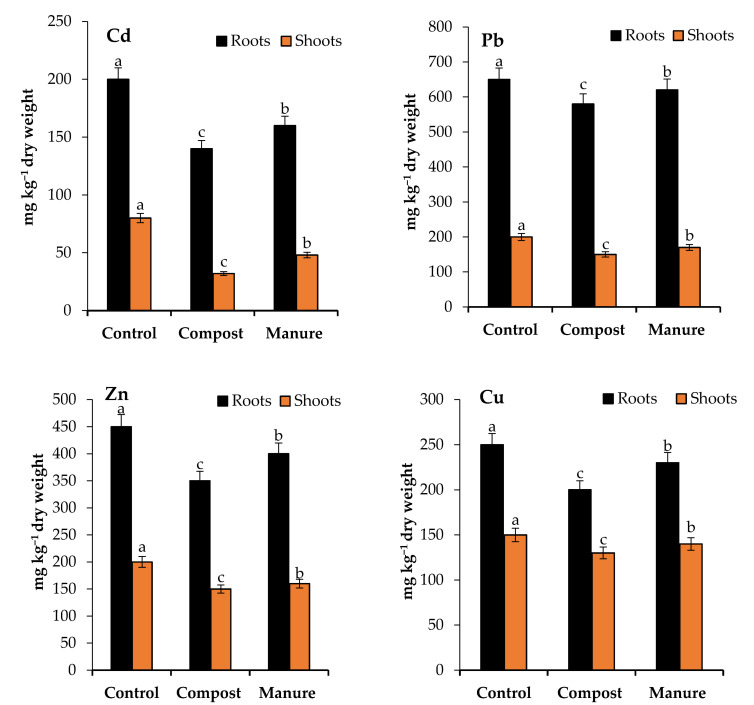
Effect of compost and manure on heavy metals content in the roots and shoots. Means (±standard deviation, *n* = 4) denoted by different letters are significantly different at *p* < 0.05.

The concentrations of cadmium (Cd), zinc (Zn), copper (Cu), and lead (Pb) in the roots and shoots were affected significantly (*p* < 0.05) by the compost and manure amendments (Figure 2). 

Compost significantly decreased Cd in the roots and shoots by 30 and 60%, while manure decreased these values by 20 and 40% compared to the control. Compost decreased Pb in the roots and shoots by 11 and 25%, while manure decreased these values by 5 and 10% compared to the control. The root and shoot concentrations of Zn were decreased by 22 and 25% as a result of compost addition in comparison with control, while in the case of manure, these concentrations decreased by 11 and 20%. The application of compost significantly decreased Cu concentration in the roots and shoots by 20 and 8%, respectively, compared to the control, while the application of manure decreased Cu concentration in the roots and shoots by 13 and 7%, compared to the control.

**Figure 3:**
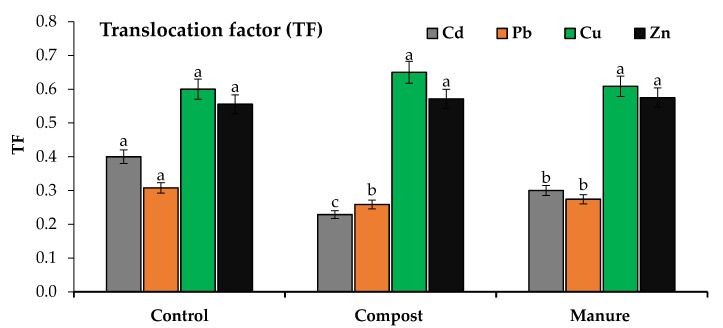
Effect of compost and manure on the translocation factor (TF) of heavy metals. Means (±standard deviation, *n* = 4) denoted by different letters are significantly different at *p* < 0.05.

**Figure 4:**
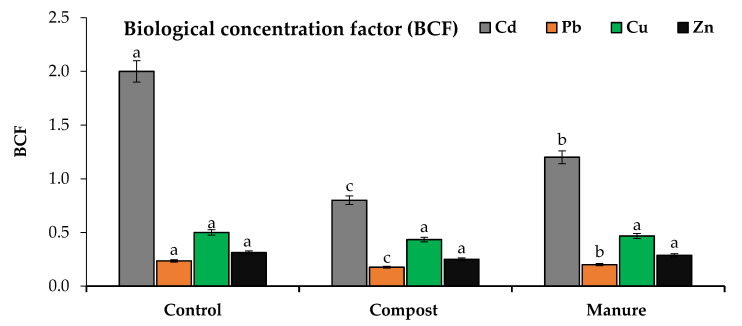
Effect of compost and manure on the biological concentration factor (BCF). Means (±standard deviation, *n* = 4) denoted by different letters are significantly different at *p* < 0.05.

The efficiency of phytoremediation can be determined by calculating BCF and TF. The translocation factor (TF) was estimated to evaluate the efficiency of compost and manure in reducing the root–shoot transfer of Cd and Pb in wavy-leaved saltbush plants. The values of TF and BCF are presented in Figure 3 and Figure 4. Compost and manure significantly decreased the Cd and Pb TF values, while the response of Zn and Cu was not significant.

Compost and manure decreased the TF values of Cd by 43 and 25% compared to the control. Compost and manure decreased the Pb TF values by 15 and 10% compared to the control. Compost and manure significantly reduced the BCF values of Cd and Pb, while the response of Zn and Cu was not significant. Compost and manure reduced the BCF values of Cd in roots by 30 and 20% and in shoots by 60 and 40% in comparison with the control. Compost and manure reduced the BCF values of Pb in roots by 11 and 5% and in shoots by 25 and 15% in comparison with the control. The efficiency of compost in reducing the TF and BCF values was greater than that of manure.

## 4. Discussion 

The accumulation of toxic elements, e.g., zinc (Zn), copper (Cu), lead (Pb), and cadmium (Cd), in agricultural lands is of great concern due to its negative impact on crop quality and productivity, food safety, and the health of soil organisms [28]. Among the heavy metals that are affecting soil and water quality, Zn, Cu, Pb, and Cd are the most dangerous elements in the ecosystem due to their high mobility in soil, plant, and water systems [29]. The studied soil contained levels of Zn, Cu, Cd, and Pb of 800, 300, 40, and 850 mg kg^−1^, respectively (Table 1). The critical values of Zn, Cu, Cd, and Pb for agricultural soils are 150–300, 50–140, 1–3, and 50–300 mg kg^−1^ [30]. The findings of this study indicate that the soil was polluted with Zn, Cu, Cd, and Pb. Pollution of soil with toxic elements causes damage in the root and shoot systems, as well as an imbalance in vital systems, e.g., it blocks the synthesis of sugars and proteins, disturbs hormone metabolism, and causes nutrients imbalance [31,32]. The threshold levels of Zn, Cu, Pb, and Cd for plants tissues are 100–400, 20–100, 30–300, and 5–30 mg kg^−1^, respectively [33]. The concentrations of Pb and Cd in the roots of the studied plants reached 580–650 and 140–200 mg kg^−1^, respectively, while in the shoots they reached 150–200 and 32–80 mg kg^−1^, respectively. Though the wavy-leaved saltbush plants grew in a soil polluted with Zn, Cu, Pb, and Cd and the concentration of these metals within the plant tissue was higher than the toxicity limits, no symptoms of toxicity by heavy metals appeared in the studied plants. This confirms that these plants tolerate high concentrations of toxic elements, whether in soil solution or in their tissues, and are thus eligible for use in the remediation of polluted soils.

The bioconcentration factor (BCF) and translocation factor (TF) are among the most important parameters to determine the potential of plants to be used in phytoremediation [34]. There are two tolerance mechanisms against metal stress according to BAF and TF values [34]. The first mechanism involves metal excluder species that have a TF value lower than 1 and accumulate toxic metals in the roots with low root–shoot transfer. The second mechanism is typical of hyperaccumulator plants that have TF and BCF values higher than 1. Based on the obtained results, wavy-leaved saltbush plants accumulate high levels of toxic metals in their roots, with TF values less than 1; therefore, the studied plants are suitable for the so-called phytostabilization technology. 

Soil characteristics, e. g., clay content, cation exchange capacity (CEC), soil pH, and organic matter (SOC), play important roles in changing heavy metals’ bioavailability [35]. The use of organic amendments is another viable option to cultivate crop in soils contaminated with Zn, Cu, Pb, and Cd [36]. The nature and quantity of organic matter in the soil affect many soil properties, e g., there is an increase in the adsorption of Zn, Cu, Pb, and Cd by soil components with increasing amounts of organic matter [37,38,39]. The addition of compost significantly affected the distribution of Cd, Zn, Cu, and Pb in soils in this study. Organic fertilizers have crucial roles in minimizing the bioavailability of Zn, Cu, Pb, and Cd in contaminated soils due to their high CEC and complexing ability [36]. In the current study, compost increased the soil CEC and SOC; on the other hand, it reduced the availability of Zn, Cu, Pb, and Cd. The decrease in the bioavailability of Zn, Cu, Pb, and Cd with the addition of organic matter was predominantly due to the effect of increasing soil CEC and SOC [38,39]. Zhou et al. [13] also stated that the application of composted organic fertilizers to alkaline lands can reduce the bioavailability and uptake of Zn and Pb. Decomposed organic fertilizers contain organic compounds that form insoluble complexes with toxic elements, which can reduce the uptake of toxic metal in plant tissues [12,14]. Compost can be used to increase the phytostabilization of Zn, Cu, Pb, and Cd in polluted lands [40]. 

Unlike compost, the addition of manure led to an increase in the bioavailability of Zn, Cu, Pb, and Cd. Manure contains relatively high levels of soluble organic matter [19]. The addition of manure reduced the soil pH to 7.52 (Table 1 and Table 3). We hypothesize that the low soil pH and the non-decomposing organic compounds in manure are the reasons for the increase in the bioavailability of Pb and Cd in the studied soil. The type of organic matter directly affects metal availability, which is very close to the degree of organic matter humification [41]. Cadmium and lead are more mobile and available when lowering the pH of moderately alkaline soils [42]. The hydroxyl and carboxyl groups in mature composted materials form stable complexes with toxic metals and reduce their bioavailability [18,19]. 

The addition of compost to metal-polluted lands increased the phytostabilization of Cd, Zn, Cu, and Pb by wavy-leaved saltbush plants. We assume that compost enhanced the phytostabilization of Zn, Cu, Pb, and Cd by reducing the bioavailability of Cd, Zn, Cu, and Pb through the formation of organo–metal complexes, minimized the metal root–shoot transfer by inducing the precipitation of Zn, Cu, Pb, and Cd in the root tissues, and mitigated the toxicity stress by enhancing photosynthesis and proline accumulation. The accumulation of malondialdehyde (MDA) in plant tissues is an indicator of oxidative stress damage due to the toxicity of Cd, Zn, Cu, and Pb in the growing medium [43,44]. Compost mitigated oxidative stress and reduced the accumulation of MDA in the leaves of wavy-leaved saltbush plants by increasing proline, which is a secondary amino acid mitigating abiotic stresses, e. g., metal stress [45]. Previous studies confirmed that some *Atriplex* plants, e. g., *A. amnicola, A. undulata*, and *A lentiformis* are able to precipitate Zn, Cu, Pb, and Cd on their root surface by forming metal–phosphate complexes [8,46,47,48,49,50]. The roots of *Atriplex* plants are characterized by their high content of phosphorous and chloride, which increases their ability in the precipitation of toxic elements on the root surface [46]. The degree of plant resistance to element toxicity depends on the type of plant and on soil conditions [48,49,50]. Therefore, improving plants’ growth conditions increases their resistance and thus improves their performance efficiency in contaminated soils [50,51,52,53,54,55,56,57].

## 5. Conclusions

Cadmium (Cd), lead (Pb), zinc (Zn), and copper (Cu) are among the most toxic elements that lead to environmental pollution and are also among the most dangerous elements to living organisms. Therefore, it is necessary to use modern technological means for the remediation of metal-polluted agricultural soils. In this pot trial, wavy-leaved saltbush plants were used to remediate an agricultural soil contaminated with Cd, Zn, Cu, and Pb by what is known as phytostabilization. The plants under study have a great ability to absorb toxic metals in their roots, with a low root–shoot transfer. The addition of compost improved the ability of the plants to reduce the availability of Cu, Zn, Cd, and Pb in the soil, while the addition of manure had an opposite effect. The application of organic fertilizers, whether as compost or manure, had positive effects in stimulating plant growth and increasing plant resistance to metals’ toxicity. Wavy-leaved saltbush plants can survive in environments that contain high concentrations of toxic metals, as they accumulate these elements in the root system, with a low transfer to the aboveground parts. More studies are needed on the extent to which these plants can be suitable in treating sewage, sediments, and contaminated water.

## Figures and Tables

**Table 1:** Basic analysis of the studied soil.

Soil Properties	Value
Texture	Sandy loam
CaCO_3_ (g kg^−1^)	60
CEC (cmol kg^−1^)	15
pH (1:2)	8.09
EC (1:2) (dS m^−1^)	2.11
Organic carbon (g kg^−1^)	3.50
Total nitrogen (mg kg^−1^)	190
Total Zn (mg kg^−1^)	800
Available Zn (mg kg^−1^)	8.2
Total Cu (mg kg^−1^)	300
Available Cu (mg kg^−1^)	2.5
Total Pb (mg kg^−1^)	850
Available Pb (mg kg^−1^)	7.0
Total Cu (mg kg^−1^)	300
Available Cu (mg kg^−1^)	2.5

**Table 2:** Chemical analysis of compost and manure.

	Organic Carbon (g kg^−1^)	Total Nitrogen (g kg^−1^)	pH 1:10	EC (dS m^−1^)	Zn (mg kg^−1^)	Cu (mg kg^−1^)	Pb (mg kg^−1^)	Cd (mg kg^−1^)
Compost	400	22	8.02	4.5	160	40	-	0.33
Manure	320	18	7.82	6.5	155	35	-	0.42

**Table 3:** Effect of cultivation, compost, and manure on some chemical characteristics of soil.

Soil Treatment	Amendments	EC (dS m^−1^)	pH (1:2)	SOC (g kg^−1^)	CEC (cmol kg^−1^)	Zn (mg kg^−1^)	Cu (mg kg^−1^)	Cd (mg kg^−1^)	Pb (mg kg^−1^)
Non-cultivatedSoil	Control	2.20 ± 0.11 ^b^	7.90 ± 0.26 ^a^	3.5 ± 0.28 ^b^	16 ± 1.5 ^b^	8.00 ± 0.32 ^b^	2.48 ± 0.18 ^b^	3.40 ± 0.15 ^b^	7.00 ± 0.28 ^b^
	Compost	3.00 ± 0.16 ^a^	7.78 ± 0.25 ^ab^	5.5 ± 0.26 ^a^	17 ± 1.8 ^ab^	6.52 ± 0.18 ^d^	2.28 ± 0.14 ^c^	3.00 ± 0.13 ^c^	6.10 ± 0.24 ^c^
	Manure	3.20 ± 0.15 ^a^	7.58 ± 0.26 ^b^	5.7 ± 0.25 ^a^	17 ± 1.7 ^ab^	8.63 ± 0.33 ^a^	2.86 ± 0.16 ^a^	4.00 ± 0.12 ^a^	8.00 ± 0.25 ^a^
Cultivated Soil	Control	2.30 ± 0.14 ^b^	7.88 ± 0.27 ^a^	3.8 ± 0.24 ^b^	16 ± 1.3 ^b^	6.54 ± 0.25 ^d^	2.00 ± 0.14 ^d^	2.55 ± 0.15 ^e^	4.70 ± 0.29 ^e^
	Compost	3.10 ± 0.18 ^a^	7.75 ± 0.24 ^ab^	5.8 ± 0.33 ^a^	18 ± 1.5 ^a^	5.86 ± 0.32 ^e^	1.76 ± 0.15 ^e^	1.96 ± 0.18 ^f^	4.00 ± 0.27 ^f^
	Manure	3.00 ± 0.11 ^a^	7.52 ± 0.22 ^b^	5.6 ± 0.42 ^a^	18 ± 1.4 ^a^	7.04 ± 0.19 ^c^	1.80 ± 0.11 ^e^	2.75 ± 0.17 ^d^	5.00 ± 0.26 ^d^
*p*		0.0001	0.008	0.005	0.002	0.009	0.005	0.001	0.004

SOC = soil organic carbon, CEC = cation exchange capacity. The available form of heavy metals was extracted by 0.005 M DTPA (diethylenetriaminepentaacetic acid). Means (±standard deviation, *n* = 4) with the same letters are not significantly different at *p* < 0.05; *p* values were determined by ANOVA.

**Table 4:** Effect of compost and manure on the growth of wavy-leaved saltbush plants.

Treatments	Plant Length (cm)	Roots Dry Weight (g pot^−1^)	Shoots Dry Weight (g pot^−1^)	Leaves Number/Plant	Leaf Area/Plant (cm^2^)
Control	130 ± 5.50 ^c^	30 ± 1.55 ^c^	100 ± 5.40 ^c^	50 ± 2.22 ^c^	100 ± 5.28 ^c^
Compost	180 ± 5.10 ^a^	45 ± 1.33 ^a^	138 ± 6.44 ^a^	80 ± 3.33 ^a^	150 ± 5.11 ^a^
Manure	165 ± 6.60 ^b^	40 ± 1.66 ^b^	125 ± 6.12 ^b^	75 ± 3.44 ^b^	138 ± 5.55 ^b^
*p*	0.01	0.001	0.003	0.01	0.002

Means (±standard deviation, *n* = 4) denoted by different letters are significantly different at *p* < 0.05. *p* values were determined by ANOVA.

## Data Availability

Data is contained within the article.
